# Household

**Published:** 1889-06

**Authors:** 


### HOUSEHOLD.

STRAWBERRIES.—At this season of the year when strawberries are plentiful, it is important to know how to make the most of them.

As FOOD.—Having a delicious saccharine,.the strawberry, when ripe, is esteemed more highly than other dessert fruits. It is both digestible and wholesome and is less liable to produce derangement of the digestive organs than any other fruit, if soft and saccharine in quality, and not indulged in too freely, or united with too much sugar and cream.

MEDICINALLY.—Strawberries for invalids and convalescents can scarcely be over-estimated. They are believed to be somewhat direutic and have been prescribed for gout and calculous diseases. The root is astringent and contains tannin. It is used in decoction for diarrhœa. The leaves are used as an herb, and tea made from them will check dysentery. In an essay delivered in 1870 before the Michigan Pomological Society by the writer of this, we find the following : "See how eagerly the children watch the first ripe strawberry, and how industriously they fill the basket with this most beautiful of all the berry family. See how the burning fever is assuaged and the parched lips refeshed by this cool and luscious fruit, infusing as it does new vigor and hope to the heart of the desponding invalid. Nor is the hope fallacious in many cases, for in the whole catalogue of simple, direct and efficacious remedies, what is there more potent to temper and purify the blood and infuse healthful exhilaration ? Linnæus was, himself, cured of the gout by this fruit, and how many have been cured since can never be told."

STRAWBERRY PYRAMID.— One pound rice, two quarts ripe, sweet strawberries to four quarts of boiling water, sprinkle gradually in the rice, after being washed. Do this so slowly that it will not stop the boiling, or if it does stop, stir now and then until the boiling is resumed. Let it boil rapidly thirty or forty minutes, then skim out and place a thin layer of the rice upon a plate, having the edges smooth. Upon this place a layer of strawberries, then another layer of rice, and so on, making each smaller in the form of a pyramid. Then finish off with a spray of berries at the top, and set it upon a larger plate decorated with strawberry leaves.

STRAWBERRIES WITH ORANGE JUICE.—Orange juice poured on strawberries, sprinkled with powdered loaf sugar.

STRAWBERRY JELLY.—Drain the juice from ripe berries, and add one pound of sugar to each pint of juice. Boil and skim the juice, and throw in the dry sugar and boil fifteen minutes.

STRAWBERRY JAM. Use fine strawberries ; add, if desired, a little fine red currant juice, then add their weight in sifted refined sugar; boil twenty minutes over a slow fire; skim as often as required, and pour into patent jelly glasses.

STRAWBERRY LILY.—Spread a layer of boiled rice on each dessert plate ; cover with strawberries, which, if ripe, need not be cooked; pour over it some sweetened strawberry juice.

